# What is the optimal systemic treatment of men with metastatic, hormone-naive prostate cancer? A STOPCAP systematic review and network meta-analysis

**DOI:** 10.1093/annonc/mdy071

**Published:** 2018-02-23

**Authors:** C L Vale, D J Fisher, I R White, J R Carpenter, S Burdett, N W Clarke, K Fizazi, G Gravis, N D James, M D Mason, M K B Parmar, L H Rydzewska, C J Sweeney, M R Spears, M R Sydes, J F Tierney

**Affiliations:** 1 MRC Clinical Trials Unit at UCL, London; 2 Salford Royal NHS Foundation Trust, Salford, UK; 3 Gustave-Roussy, University of Paris Sud, Villejuif; 4 Department of Medical Oncology, Institut Paoli Calmettes, Marseille, France; 5 Institute of Cancer and Genomic Sciences, University of Birmingham, Birmingham; 6 Queen Elizabeth Hospital, Birmingham; 7 School of Medicine, Cardiff University, Cardiff, UK; 8 Dana-Farber Cancer Institute, Harvard Medical School, Boston, USA

**Keywords:** prostate cancer, abiraterone, docetaxel, systematic review, network meta-analysis, androgen-deprivation therapy

## Abstract

**Background:**

Our prior Systemic Treatment Options for Cancer of the Prostate systematic reviews showed improved survival for men with metastatic hormone-naive prostate cancer when abiraterone acetate plus prednisolone/prednisone (AAP) or docetaxel (Doc), but not zoledronic acid (ZA), were added to androgen-deprivation therapy (ADT). Trial evidence also suggests a benefit of combining celecoxib (Cel) with ZA and ADT. To establish the optimal treatments, a network meta-analysis (NMA) was carried out based on aggregate data (AD) from all available studies.

**Methods:**

Overall survival (OS) and failure-free survival data from completed Systemic Treatment Options for Cancer of the Prostate reviews of Doc, ZA and AAP and from recent trials of ZA and Cel contributed to this comprehensive AD-NMA. The primary outcome was OS. Correlations between treatment comparisons within one multi-arm, multi-stage trial were estimated from control-arm event counts. Network consistency and a common heterogeneity variance were assumed.

**Results:**

We identified 10 completed trials which had closed to recruitment, and one trial in which recruitment was ongoing, as eligible for inclusion. Results are based on six trials including 6204 men (97% of men randomised in all completed trials). Network estimates of effects on OS were consistent with reported comparisons with ADT alone for AAP [hazard ration (HR) = 0.61, 95% confidence interval (CI) 0.53–0.71], Doc (HR = 0.77, 95% CI 0.68–0.87), ZA + Cel (HR = 0.78, 95% CI 0.62–0.97), ZA + Doc (HR = 0.79, 95% CI 0.66–0.94), Cel (HR = 0.94 95% CI 0.75–1.17) and ZA (HR = 0.90 95% CI 0.79–1.03). The effect of ZA + Cel is consistent with the additive effects of the individual treatments. Results suggest that AAP has the highest probability of being the most effective treatment both for OS (94% probability) and failure-free survival (100% probability). Doc was the second-best treatment of OS (35% probability).

**Conclusions:**

Uniquely, we have included all available results and appropriately accounted for inclusion of multi-arm, multi-stage trials in this AD-NMA. Our results support the use of AAP or Doc with ADT in men with metastatic hormone-naive prostate cancer. AAP appears to be the most effective treatment, but it is not clear to what extent and whether this is due to a true increased benefit with AAP or the variable features of the individual trials. To fully account for patient variability across trials, changes in prognosis or treatment effects over time and the potential impact of treatment on progression, a network meta-analysis based on individual participant data is in development.

Key Message

Our results show that adding AAP to ADT is more effective than doc plus ADT in improving survival in mHNPC, however, the absolute difference in survival benefit between the two treatments remains uncertain. A STOPCAP network meta-analysis using individual participant data is underway to resolve uncertainty and to assess how trial or patient variability impacts on these results.

## Introduction

Numerous randomised controlled trials (RCTs) have evaluated or are currently evaluating, the addition of other therapies to androgen-deprivation therapy (ADT) in men with metastatic hormone-naive prostate cancer (mHNPC). To determine reliably which are effective, we are conducting a series of systematic reviews under the auspices of the Systemic Treatment Options for Cancer of the Prostate (STOPCAP) collaboration. Our prior STOPCAP systematic reviews showed improved survival when abiraterone acetate plus prednisolone/prednisone (AAP) or docetaxel (Doc), but not zoledronic acid (ZA), were added to ADT [1, 2]. Trial evidence also suggests a benefit of combining celecoxib (Cel) with ZA and ADT [3].

However, only the reported comparisons of the STAMPEDE multi-arm, multi-stage (MAMS) platform and the ongoing Prostate Cancer Consortium in Europe (PEACE)-1 trial (NCT01957436) will be able to provide head-to head results comparing these therapies. Network meta-analysis [4, 5], which takes advantage of both direct and indirect comparisons, is therefore needed to determine reliably which is the optimal treatment(s), and so to inform patients, clinicians and policy makers. We have therefore conducted a systematic review and network meta-analysis of aggregate data (AD) to assess the optimal systemic treatments for men with mHNPC, making use of existing STOPCAP reviews [1, 2] and up-to-date results from individual trials, and also taking account of the MAMS platform design of the STAMPEDE trial protocol.

## Methods

The full protocol for this review was registered in July 2017 (http://www.crd.york.ac.uk/PROSPERO/display_record.asp? ID=CRD42017071811, 1 March 2018, date last accessed). 

### Eligibility criteria

The eligibility criteria for inclusion in this network meta-analysis mirror those in prior systematic reviews [1, 2]. In brief, eligible trials should have been randomised in a way which precluded prior knowledge of the treatment assigned and compared ADT alone with ADT in combination with any of the agents (or combinations of agents) under consideration, namely celecoxib (Cel), zoledronic acid (ZA), celecoxib and zoledronic acid (ZA + Cel), docetaxel (Doc), zoledronic acid + docetaxel (ZA + Doc) or abiraterone acetate plus prednisolone (AAP). The men randomised were diagnosed with mHNPC, and either starting or responding to the first-line ADT for metastatic disease (they may have received prior treatments for early, localised disease). Trials were also eligible if they met the above criteria but additionally co-administered supportive treatments on the experimental arm only. Trials were excluded if they had randomised men who had castration-resistant prostate cancer or if they had included additional first-line treatments only on the control arm only.

### Trial identification

As part of the wider STOPCAP project, we regularly and systematically search a number of sources to identify all published, unpublished and ongoing trials in mHNPC, providing a comprehensive and up-to-date database of all RCTs eligible for all of our STOPCAP systematic reviews. We also request regular updates from relevant trial teams on the status and reporting plans. Thus, all trials included in our previous STOPCAP reviews [1, 2] and any additional RCTs meeting the eligibility criteria were included.

In summary, we searched MEDLINE, EMBASE, clinicaltrials.gov and the Cochrane Central Register of Controlled Trials (CENTRAL), using database-specific search strategies. We also searched proceedings from relevant conferences. In addition, reference lists of review articles and bibliographies of identified trial reports were screened for further eligible trials. Full search strategies have been previously reported [1, 2].

### Outcomes

The primary outcome was overall survival (OS), with failure-free survival (FFS) the secondary outcome.

### Data extraction

The principal data extracted or derived from included studies was the log hazard ratio and SE or the information to estimate them [e.g. a hazard ratio (HR) and confidence interval (CI) or *P* value [6] for OS and FFS]. Outcome definitions were also extracted for each trial to ensure their consistency and the appropriateness of combining results in a formal meta-analysis. Additional summary data including start and end dates of recruitment, details of treatment schedules on the control and experimental arms, numbers of patients and their demographics were also extracted, either directly from the trial publications or from prior systematic reviews.

### Assessing the risk of bias of included trials

Assessment of study quality for all trials included in the prior STOPCAP reviews was previously carried out in the individual reviews, using the Cochrane risk of bias tool [7] and all included studies were assessed as having low risk of bias based on reported information and study protocols [8]. Risk of bias assessments for additional eligible studies identified for inclusion in the network meta-analysis was also carried out using the Cochrane tool.

### Analysis

One of the trials identified as eligible for inclusion, the STAMPEDE trial, used a MAMS platform design. The nature of this type of design means that some patients randomised to the control arm contribute to multiple pairwise treatment comparisons within the trial. Therefore, to appropriately include data from this trial in an AD network meta-analysis, we needed to assess the correlations between the effect estimates arising due to periods of overlap in the common control arm. Correlations were estimated using the control-arm event counts within the periods of overlap for which data were obtained directly from the STAMPEDE investigators.

The primary analysis was carried out using a frequentist contrast-based network meta-analysis model and the network suite of commands [5] in Stata v14.1 (StataCorp, Texas, USA). Because all trials in this network, apart from the MAMS trial, are two-arm comparisons with the common control arm, any test for inconsistency would assess heterogeneity between the MAMS and other studies. Therefore, we fitted a consistency model and assessed heterogeneity, assuming a common heterogeneity variance across all comparisons. In the primary analysis, all comparisons of treatment combinations (e.g. ADT + ZA + Doc versus ADT) were assumed to be unrelated to the comparisons of their component treatments (e.g. ZA and Doc). A sensitivity analysis assumed additive treatment effects.

**Figure 1.**
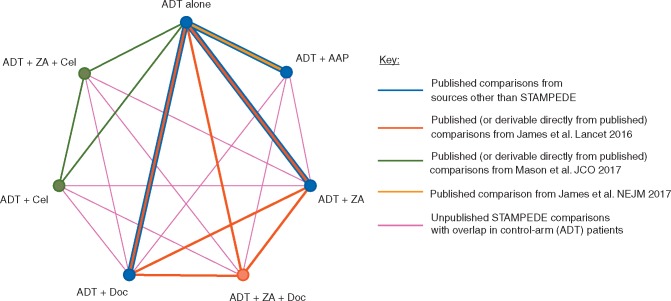
Network meta-analysis structure. AAP, abiraterone acetate plus prednisolone/prednisone; ADT, androgen-deprivation therapy; Cel, celecoxib; Doc, docetaxel; ZA, zoledronic acid.

A network map (Figure 1) was constructed to display all of the available relationships, with distinct treatments represented by nodes, and trials (or separate trial comparisons within the single MAMS design trial) by lines joining appropriate nodes. The thickness of the lines, representing the extent of available data for each comparison, was estimated from the combined number of events for all trials contributing to each individual comparison. Borrowing of strength statistics, which represent the proportion of the information for each treatment comparison that the indirect evidence from the network model has contributed, were calculated using the score decomposition method [9].

Estimates of relative effect for each pairwise treatment comparison from the primary consistency model were estimated on the HR scale along with corresponding 95% confidence limits and displayed in a network forest plot [5]. Treatment rankings were also calculated and summarised as a surface under the cumulative rank (SUCRA) value, representing the re-scaled mean ranking [10]. Further detailed methods relating to all the planned analyses may be found in the Statistical Analysis Plan (available on request).

Finally, we conducted an indirect comparison of the two most effective treatments to estimate the relative difference in the size of the effects. We estimated the absolute benefit of treatment on OS at 3 years by applying the HR estimates to the approximate survival at 3 years.

### Role of the funding source

The funders of the study (Medical Research Council and Prostate Cancer UK) had no role in study design, data collection, data analysis, data interpretation or writing of the report. The corresponding author had full access to all the data in the study and had final responsibility for the decision to submit for publication.

## Results

### Description of the included trials

Searches undertaken for the STOPCAP project identified 11 trials that were eligible for inclusion in the network meta-analysis. Five of these 11 eligible trials could not be included in the network meta-analysis. Of these five, two trials that together randomised 72 men to receive ADT or ADT plus doc and two trials that together randomised 102 men to receive ADT versus ADT plus ZA identified as eligible for a previous STOPCAP review [2] could not be included here as they had not yet presented results for survival outcomes. The fifth trial, PEACE-1 (NCT01957436), comparing standard of care with or without AAP continues to recruit towards a target of 1168 men and no results are currently available for inclusion.

Therefore, six RCTs that had reported results were included in the network meta-analysis (Figure 1, Table 1). Two trials compared ADT with ADT plus ZA; two compared ADT with ADT plus Doc and one trial compared ADT with ADT plus AAP. The final trial, STAMPEDE [11], contributed six separate treatment comparisons [3, 12, 13] to the network. Four comparisons were of ADT with Cel, ZA, Doc or AAP and two further comparisons were of ADT with combinations of ZA plus Cel or ZA plus Doc. Importantly, although each of the six comparisons shared a common control arm, there was some non-contemporaneous recruitment to individual treatment comparisons. In total, 6204 men were included in the network meta-analysis, representing 97% of men randomised in all completed eligible trials (at least 83% of men randomised in all 11 eligible trials). Accounting for the shared control arm patients in the STAMPEDE trial, 2615 men were randomised to receive ADT alone, and 3589 men were randomised to receive ADT in combination with one of the treatments being considered in the network.

**Table 1.** Description of included trials (or treatment comparisons from the STAMPEDE trial) and FFS definition used in the trial. All trials had a control arm of ADT

Trial	Recruitment period	Median follow-up (months)	Treatment	Treatment (*N*)	Control (*N*)	Definition of FFS
CALGB 90202 [21]	June 2004 to April 2012	Unknown	ADT + ZA	323	322	Time to first bone progression, PSA progression, or death
GETUG 15 [22]	Oct 2004 to Dec 2008	84	ADT + Doc	192	193	Time to PSA progression, clinical progression or death
STAMPEDE (Arms A versus D) [3]	Oct 2005 to April 2011	69	ADT + Cel	188	377	Time to PSA failure, progression of local, lymph-node, or distant metastases; or death from prostate cancer
STAMPEDE (Arms A versus F) [3]	Oct 2005 to April 2011	69	ADT + ZA + Cel	190	377	Time to PSA failure, progression of local, lymph-node, or distant metastases; or death from prostate cancer
STAMPEDE (Arms A versus B) [13]	Oct 2005 to March 2013	43	ADT +ZA	366	724	Time to PSA failure, progression of local, lymph-node, or distant metastases; or death from prostate cancer
STAMPEDE (Arms A versus C) [13]	Oct 2005 to March 2013	43	ADT + Doc	362	724	Time to PSA failure, progression of local, lymph-node, or distant metastases; or death from prostate cancer
STAMPEDE (Arms A versus E) [13]	Oct 2005 to March 2013	43	ADT + ZA + Doc	365	724	Time to PSA failure, progression of local, lymph-node, or distant metastases; or death from prostate cancer
CHAARTED [23]	July 2006 to Dec 2012	54	ADT + Doc	397	393	Time to PSA rise or clinical progression
ZAPCA (KYUH TRIG0705) [24]	May 2008 to Dec 2010	42	ADT +ZA	109	110	Time to earliest date of PSA progression, clinical progression, first SRE, death for any reason, or cessation of protocol treatment for any reason
STAMPEDE (Arms A versus G) [12]	Nov 2011- Jan 2014	40	ADT + AAP	500	502	Time to PSA failure, progression of local, lymph-node, or distant metastases; or death from prostate cancer
LATITUDE [14]	Feb 2013 to Dec 2014	30	ADT + AAP	597	602	Time to radiographic progression or death from any cause

AAP, abiraterone acetate plus prednisolone/prednisone; ADT, androgen-deprivation therapy; Cel, celecoxib; Doc, docetaxel; FFS, failure-free survival; SRE, skeletal related events; ZA, zoledronic acid.

OS and FFS were reported for all of the treatment comparisons. The definition of FFS included time to prostate specific antigen (PSA) or clinical progressionor death for all of the trials, except LATITUDE [14], which did not include time to PSA progression and CHAARTED [15], which did not include time to death. Further details of the trials, including the definitions used for FFS, are given in Table 1.

### Borrowing of strength from the network

Inclusion in the network led to a gain in information for each of the pairwise treatment comparisons (Table 2). For OS, this ranged from 0.9% (AAP) to 9.2% (ZA + Doc). For FFS, the gains were generally greater and ranged from 6.7% (Doc) to 21.7% (ZA + Doc).

**Table 2.** Borrowing of strength statistics (% of information gained per pairwise analysis through inclusion in the network)

Comparison	OS, %	FFS
ADT versus ADT +Cel	5.4	17.3
ADT versus ADT + ZA	3.9	7.7
ADT versus ADT + ZA + Cel	5.2	17.1
ADT versus ADT + Doc	2.0	6.7
ADT versus ADT + ZA + Doc	9.2	21.7
ADT versus ADT + AAP	0.9	7.4

AAP, abiraterone acetate plus prednisolone/prednisone; ADT, androgen-deprivation therapy; Cel, celecoxib; Doc, docetaxel; FFS, failure-free survival; OS, overall survival; ZA, zoledronic acid.

### Overall survival

**Figure 2.**
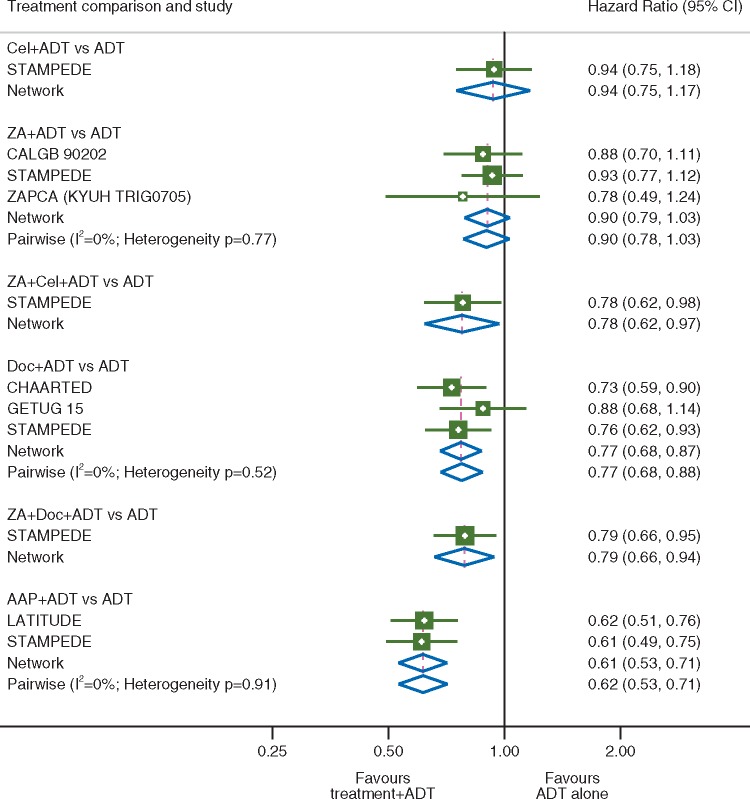
Overall survival. Forest plot of network and pairwise estimates of treatment effects [all treatments compared with androgen-deprivation therapy (ADT) alone]. AAP, abiraterone acetate plus prednisolone/prednisone; CI, confidence interval; Cel, celecoxib; Doc, docetaxel; ZA, zoledronic acid.

The network meta-analysis HR estimates suggested that compared with ADT alone each of AAP (HR = 0.61, 95% CI 0.53–0.71), Doc (HR = 0.77, 95% CI 0.68–0.87), ZA + Doc (HR = 0.79, 95% CI 0.66–0.94) and ZA + Cel (HR = 0.78, 95% CI 0.62–0.97) in combination with ADT improved survival. There was no survival advantage observed with ADT in combination with either ZA (HR = 0.90, 95% CI 0.79–1.03) or Cel (0.94, 95% CI 0.75–1.17) over ADT alone. For the comparisons of ADT versus ADT + Cel, ADT + ZA + Cel and ADT + ZA + Doc, the only data available were from single comparisons within the STAMPEDE trial [3, 13]. There was no evidence of heterogeneity between the effects of treatment within any of the individual treatment comparisons and all of the estimates from the network analysis were in keeping with those obtained in the previously reported pairwise meta-analyses where available (Figure 2).

### Treatment rankings

When used in combination with ADT, AAP has the highest probability (94%) of being the most effective treatment, Doc has a 35% probability of being the second-best treatment and ADT alone has the highest probability of being the least effective treatment (67%, Table 3).

**Table 3.** Treatment ranking (% probability) and SUCRA values based on overall survival results

	AAP	Doc	ZA + Doc	ZA + Cel	ZA	Cel	ADT alone
Best	94.2	0.7	1.3	3.8	0.0	0.0	0.0
Second best	5.3	34.9	25.5	33.0	0.3	1.0	0.0
Third best	0.4	36.8	30.3	27.0	2.4	3.1	0.0
Fourth best	0.1	23.6	30.8	23.9	12.2	9.3	0.1
Fifth best	0.0	3.8	9.3	9.3	48.7	26.0	2.9
Sixth best	0.0	0.2	2.6	2.5	31.3	33.6	29.8
Worst	0.0	0.0	0.2	0.5	5.1	27.0	67.2
SUCRA	1.0	0.7	0.6	0.6	0.3	0.2	0.1

AAP, abiraterone acetate plus prednisolone/prednisone; ADT, androgen-deprivation therapy; Cel, celecoxib; Doc, docetaxel; SUCRA, surface under the cumulative rank; ZA, zoledronic acid.

### Failure-free survival

**Figure 3.**
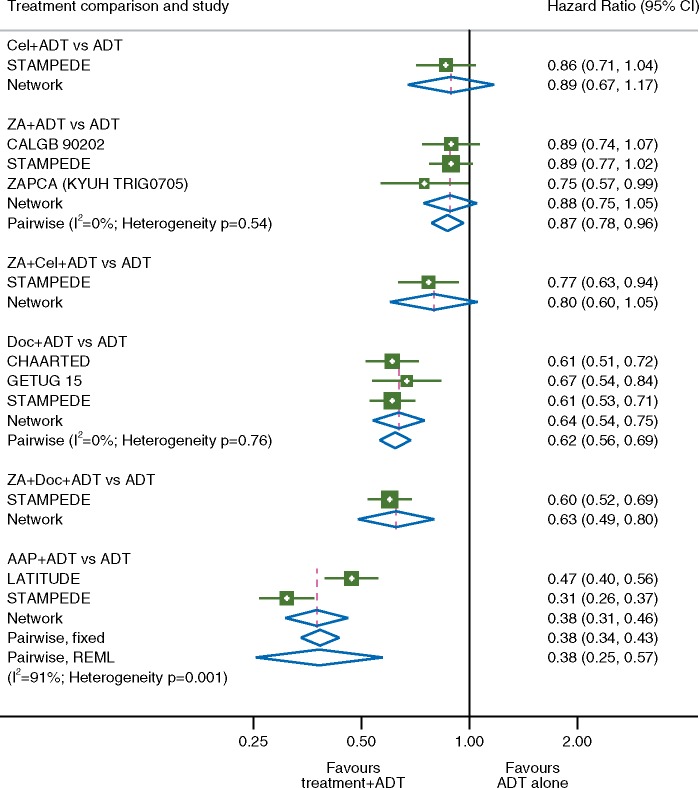
Failure-free survival. Forest plot of network and pairwise estimates of treatment effects [all treatments compared with androgen-deprivation therapy (ADT) alone]. AAP, abiraterone acetate plus prednisolone/prednisone; CI, confidence interval; Cel, celecoxib; Doc, docetaxel; ZA, zoledronic acid.

There was an FFS benefit associated with adding ADT to each of AAP (HR = 0.38 95% CI 0.31–0.46), Doc (HR + 0.64 95% CI 0.54–0.75) and ZA + Doc (HR = 0.63 95% CI 0.49–0.80) compared with ADT alone. No statistically significant benefit was seen with the addition of Cel (HR = 0.89 95% CI 0.67–1.17); ZA + Cel (HR = 0.80 95% CI 0.60–1.05) or ZA alone (HR = 0.88 95% CI 0.75–1.05). In all cases, the HR estimates obtained through the network were very similar to those obtained using a standard pairwise meta-analysis, providing confirmation that the network model is behaving as expected. There was evidence of variation or inconsistency between the effects of treatment within the individual treatment comparisons of ADT versus ADT plus AAP (*I*^2^=91%, heterogeneity *P* = 0.001) where there was a large variation between the size of the relative effects (but not the direction of the effect) observed between the two included trial comparisons. However, there was no evidence of variation or inconsistency between the effects of treatment within the remaining treatment comparisons, and all of the estimates from the network analysis were in keeping with those obtained in the previously reported pairwise meta-analyses where available (Figure 3).

Therefore, we carried out a sensitivity analysis using the outcome of time to PSA failure as reported in LATITUDE to assess the robustness of our primary analysis. This analysis, whilst not changing our interpretation, did result in an HR estimate from the network analysis was slightly more in favour of treatment (HR = 0.30, 95% CI 0.27–0.34) with no evidence of variation of inconsistency (*I*^2^=0, heterogeneity *P* = 0.78).

Based on the treatment rankings, when combined with ADT, AAP has the highest probability (100%) of being the most effective treatment in terms of FFS, whilst either Doc alone (45% probability) or in combination with ZA (52% probability) is most likely to be the second-best treatment. ADT alone has the highest probability of being the least effective treatment (73%, Table 4).

**Table 4.** Treatment ranking (% probability) and SUCRA values based on failure-free survival results

	AAP	ZA + Doc	Doc	ZA + Cel	ZA	Cel	ADT alone
Best	100.0	0.0	0.0	0.0	0.0	0.0	0.0
Second best	0.0	52.0	45.1	2.6	0.0	0.3	0.0
Third best	0.0	41.3	47.9	9.5	0.1	1.2	0.0
Fourth best	0.0	5.7	6.7	53.3	14.7	19.1	0.5
Fifth best	0.0	1.0	0.3	21.5	42.0	31.4	3.8
Sixth best	0.0	0.0	0.0	10.4	37.6	29.1	22.9
Worst	0.0	0.0	0.0	2.7	5.6	18.9	72.8
SUCRA	1.0	0.7	0.7	0.4	0.3	0.3	0.1

AAP, abiraterone acetate plus prednisolone/prednisone; ADT, androgen-deprivation therapy; Cel, celecoxib; Doc, docetaxel; SUCRA, surface under the cumulative rank; ZA, zoledronic acid.

### Indirect comparison of the two most effective treatments

**Figure 4.**
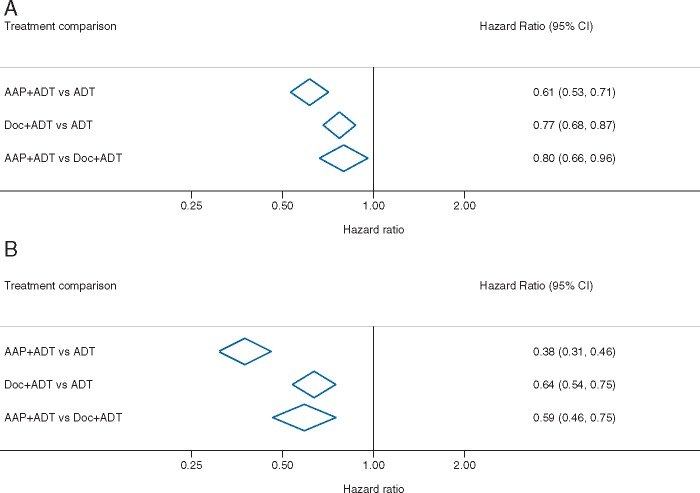
Indirect comparison of the two most effective treatment combinations (A) overall survival and (B) failure-free survival. AAP, abiraterone acetate plus prednisolone/prednisone; ADT, androgen-deprivation therapy; CI, confidence interval; Cel, celecoxib; Doc, docetaxel; ZA, zoledronic acid.

When used in combination with ADT, two treatments, AAP and Doc, emerged as being effective in terms of improving both OS and FFS relative to ADT alone, and with the greatest probabilities of being the top two most effective treatments; therefore, they were compared indirectly in a pairwise comparison. The HR estimate for the effect of ADT + AAP relative to the effect of ADT + Doc on OS is 0.80 (95% CI 0.66–0.96). Assuming a baseline OS of 60% at 3 years with ADT + Doc, this translates to an absolute survival benefit associated with AAP of 6% (95% CI = 1% to 11%), that is, to 66% at 3 years (95% CI 61% to 71%). For FFS, the HR for the effect of ADT + AAP relative to ADT + Doc is 0.59 (95% CI 0.46–0.75) (Figure 4).

## Discussion

### Summary of results

Based on the current data, either AAP or Doc alongside ADT improves the survival of men with mHNPC. Although AAP has the highest probability of being the most effective treatment, with Doc + ADT the second most effective treatment, uncertainty remains about the difference in absolute magnitude of survival benefit between these two treatments (between 1% and 9% at 3 years). Adding ZA + Doc or ZA + Cel to ADT also improved survival relative to ADT alone.

### Strengths

This is the first network meta-analysis to comprehensively assess and rank the effects of all the systemic treatments for mHNPC recently tested in RCTs. By working with trial investigators, we have been able to make use of the most up-to-date, and consistent AD from six trials and 6204 men, representing 97% of those randomised in reported, eligible trials. Thus, it provides the most reliable assessment of the relative effects of these agents to date. In particular, our analysis has been able to shed light on the comparison of AAP and Doc in conjunction with ADT and has also reinforced the observed survival benefit associated with the combination of Cel and ZA, although not with either of these agents given individually [3]. Furthermore, to the best of our knowledge, this is the first example of a network meta-analysis that has taken account of the complexities of including a MAMS platform trial, appropriately adjusting the analysis for the proportion of the control-arm patients common to different pairwise comparisons. Obtaining limited unpublished information direct from the investigators made this possible.

### Limitations

Whilst we have been comprehensive in our approach to this review, relying on AD inevitably has limitations. For example, whilst most trials include PSA progression as part of the definition of FFS, the LATITUDE trial [14] presented results for PSA failure and clinical or radiological progression separately. Whilst our pre-specified primary analysis used time to clinical or radiological progression for the LATITUDE trial, a sensitivity analysis which used time to PSA failure data from LATITUDE showed that our results are robust to these different definitions. In hindsight, although this might have been the preferable primary analysis, it would have made little difference to our interpretation and conclusions.

There are differences in the patient characteristics, which may influence the effects of the various treatments either within or across trials. Despite these differences, however, there has been no clear evidence of heterogeneity in the effects of zoledronic acid, docetaxel or abiraterone across trials in the individual STOPCAP systematic reviews [1, 2]. Nevertheless, some of the individual trials have suggested treatment–covariate interactions, notably that the effects of Doc may be moderated by the extent of metastatic disease volume and that the effects of AAP may be associated with age [1, 12]. There are no well-defined methods to appropriately assess such interactions in an AD network meta-analysis. Therefore, they will be best investigated through the collection and re-analysis of individual participant data from all of the eligible trials, which the STOPCAP collaborators are currently working to undertake.

### Context

Previously, both Doc and AAP have been shown to improve survival and delay progression in men with mHNPC [2, 16]. However, as no trials have set out to directly compare ADT plus docetaxel with ADT plus AAP, network meta-analysis provides the only comprehensive approach to this treatment comparison. However, the design of the STAMPEDE trial, in which treatments were simultaneously compared against ADT alone, has enabled an opportunistic analysis in which outcomes for men randomised within the same time frame to receive ADT plus Doc or ADT plus AAP were compared [17]. Whilst not a fully powered analysis, the results demonstrated an advantage of AAP over Doc with respect to FFS (HR = 0.51, 95% CI 0.39–0.67, *P* < 0.001), which did not translate to a survival benefit (HR = 1.16, 95% CI 0.82–1.65, *P* = 0.40). It is not clear why the findings of the STAMPEDE analysis differ with respect to OS from the findings of the network meta-analysis. The STAMPEDE analysis is a direct comparison of men who were randomised contemporaneously to receive either AAP or Doc in addition to ADT. The network meta-analysis has considerably more power to detect differences in treatment effects, due to both the inclusion of more trials and more men, and the additional strength that other treatment comparisons lend within the network. However, the inclusion of more trials, that randomised men to receive a variety of treatments over a longer time period inevitably brings with it greater variability, due to the broader case mix of patients, with different prognoses, and with differing access to treatments after progression of disease. It has not been possible in this analysis to account for changes in care over time, particularly whilst the number of new treatments available at relapse has rapidly increased, and once again this is best achieved through the collection and analysis of individual participant data.

Other network meta-analyses have been reported. Feyerabend et al. [18] presented the results of an indirect comparison of AAP and Doc based on the results of just two trials. Wallis et al. [19] have also reported indirect comparisons of ADT plus AAP with ADT plus Doc. However, we have concerns regarding the methodological approaches used by the authors, in particular, the inclusion of patients with and without metastases; the double-counting of shared control arm patients, and therefore correlations, from the STAMPEDE trial and the use of flawed subgroup analysis methodology [20].

Finally, a key question that remains for both clinicians and patients is whether the two most effective treatments, AAP and Doc, could be safely combined and if so, what the impact of the combination may be on OS. This question will only be resolved when results from the ongoing PEACE-1 trial are available.

### Conclusions

Our results support the use of either AAP or Doc alongside ADT in men with mHNPC. AAP appears to be the most effective treatment, but it is not clear to what extent and whether this is due to a true increased benefit with AAP or to the variable features of the individual trials. To fully account for patient variability across trials, changes in prognosis or treatment effects over time, and the potential impact of treatment on progression, a network meta-analysis based on individual participant data is currently in development.
